# Dark diversity reveals importance of biotic resources and competition for plant diversity across habitats

**DOI:** 10.1002/ece3.6351

**Published:** 2020-06-04

**Authors:** Camilla Fløjgaard, Jose W. Valdez, Lars Dalby, Jesper Erenskjold Moeslund, Kevin K. Clausen, Rasmus Ejrnæs, Meelis Pärtel, Ane Kirstine Brunbjerg

**Affiliations:** ^1^ Department of Bioscience – Kalø Aarhus University Rønde Denmark; ^2^ Department of Botany Institute of Ecology and Earth Sciences University of Tartu Tartu Estonia

**Keywords:** Beal's smoothing, community completeness, conservation, regional species pool, species co‐occurrence, vascular plants

## Abstract

Species richness is the most commonly used metric to quantify biodiversity. However, examining dark diversity, the group of missing species which can potentially inhabit a site, can provide a more thorough understanding of the processes influencing observed biodiversity and help evaluate the restoration potential of local habitats. So far, dark diversity has mainly been studied for specific habitats or large‐scale landscapes, while less attention has been given to variation across broad environmental gradients or as a result of local conditions and biotic interactions. In this study, we investigate the importance of local environmental conditions in determining dark diversity and observed richness in plant communities across broad environmental gradients. Using the ecospace concept, we investigate how these biodiversity measures relate to abiotic gradients (defined as position), availability of biotic resources (defined as expansion), spatiotemporal extent of habitats (defined as continuity), and species interactions through competition. Position variables were important for both observed diversity and dark diversity, some with quadratic relationships, for example, plant richness showing a unimodal response to soil fertility corresponding to the intermediate productivity hypothesis. Interspecific competition represented by community mean Grime C had a negative effect on plant species richness. Besides position‐related variables, organic carbon was the most important variable for dark diversity, indicating that in late‐succession habitats such as forests and shrubs, dark diversity is generally low. The importance of highly competitive species indicates that intermediate disturbance, such as grazing, may facilitate higher species richness and lower dark diversity.

## Graphical abstract

Dark diversity, the group of missing species that can potentially inhabit a site, is strongly related to abiotic conditions or habitat types. Dark diversity is positively correlated with competition estimated using community mean Grime C and negatively correlated with soil organic carbon indicating that dark diversity increases at sites dominated by few, but abundant competitive species, but is reduced in undisturbed sites with long‐term stability.

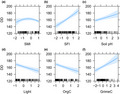

## 1 INTRODUCTION

The global biodiversity crisis represents one of the most critical challenges in the 21st century (Butchart et al., 2010; Díaz et al., 2019; Tittensor et al., 2014). Achieving conservation goals and prioritizing efforts requires appropriate metrics to quantify biodiversity and identify the factors driving the declines. The most commonly used measure is observed species richness which traditionally depends on field surveys to count the individual species. Although observed diversity can provide valuable insights into the richness of species within a given site, it does not account for the absent part of the species pool that could potentially inhabit that site considering suitable environmental conditions and biogeographic history, that is, the dark diversity (Pärtel, Szava‐Kovats, & Zobel, 2011). Identifying this part of the biodiversity can provide a more thorough understanding of the processes influencing biodiversity and help evaluate the restoration potential of local habitats (Lewis, Szava‐Kovats, Pärtel, & Evolution, 2016).

In contrast to observed diversity, dark diversity focuses on the portion of diversity potentially able to occur in a particular habitat type, but which is currently missing. The ultimate potential of biodiversity at a given site is mainly determined by large‐scale biogeographic and evolutionary processes (i.e., species diversification and historic migration patterns) which create the set of species that can theoretically inhabit a site, defined as the species pool (Cornell & Harrison, 2014; Pärtel, Zobel, Zobel, van der Maarel, & Partel, 1996; Zobel, 2016). Although this species pool influences biodiversity patterns, the observed species are filtered by local processes such as biotic interactions, population dynamics, dispersal, anthropogenic disturbance, and stochastic events (Cornell & Harrison, 2014; Pärtel, Szava‐Kovats, & Zobel, 2013; Ronk, Szava‐Kovats, & Pärtel, 2015; Zobel, 2016). Dark diversity therefore reconciles the role of local (biotic interactions, abiotic filters, dispersal, stochastic events) and large‐scale processes (species diversification and historic migration patterns) underlying biodiversity patterns and biological communities (Pärtel, 2014; Pärtel et al., 2011). This metric can provide insight into the determinants of missing species by helping us understand what characterizes species that are often missing or sites missing many species. Quantifying dark diversity patterns, in combination with observed diversity patterns, can allow researchers to better understand the mechanisms and processes acting on individual populations or entire communities (Pärtel, Öpik, et al., 2017).

The potential value of dark diversity to guide conservation and restoration planning has been demonstrated for mammals (Estrada, Márcia Barbosa, & Real, 2018), sharks (Boussarie et al., 2018), and fungi (Pärtel, Öpik, et al., 2017; Pärtel, Zobel, Öpik, & Tedersoo, 2017), but most studies have considered plants (Bennett et al., 2016; Moeslund et al., 2017; Ronk, De Bello, Fibich, & Pärtel, 2016; Ronk et al., 2015). Dark diversity has also proven valuable in understanding plant diversity patterns, such as determining that vascular plant dark diversity across Europe follows a latitudinal gradient (Ronk et al., 2015). So far, the plant traits likely to increase a species’ probability of being part of the dark diversity include stress intolerance, height, adaptation to low light and nutrient levels, and producing fewer and heavier seeds (Moeslund et al., 2017; Riibak et al., 2015; Riibak, Ronk, Kattge, & Pärtel, 2017). Moreover, understanding the ecological processes governing plant dark diversity contributes to understanding biodiversity in general, that is, plants are bioindicators of their abiotic environment and anthropogenic impact (Bartelheimer & Poschlod, 2016), and they form the living and dead organic carbon pools and biotic surfaces that are the niche space for all other taxonomic groups (Brunbjerg et al., 2017; DeAngelis, 2012), and vascular plants predict biodiversity across environmental gradients and broad taxonomic realms, and are related to the occurrence of regionally red‐listed species of other taxa (Brunbjerg et al., 2018). Nevertheless, as a relatively new concept, more research is required to establish its full potential and to understand the ecological processes governing dark diversity across plant communities.

Most dark diversity research has ignored the variability between types of habitats and have mostly been restricted to narrow sets of variables and specific habitats (Riibak et al., 2015) or large‐scale landscapes (Ronk et al., 2015, 2016), with no studies examining how dark diversity varies across large environmental gradients or the importance of local conditions and biotic interactions. Applying dark diversity within one habitat type may produce adequate results, for example, as seen for grasslands (Riibak et al., 2015), but biodiversity varies greatly across ecosystems and is highly dependent on the habitat and region of interest (Bello et al., 2016). Dark diversity can be used to derive community completeness, a relativized biodiversity index, which has been proposed as a valuable tool for facilitating biodiversity comparisons irrespective of regions, ecosystems, and taxonomic groups (Pärtel et al., 2013). The community completeness index can be defined, in general terms, as the proportion of species from the regional species pool which have dispersed to and established at a site after abiotic and biotic filtering (Pärtel et al., 2013). Since patterns in observed species richness may mimic patterns in dark diversity (e.g., exhibit a strong latitudinal gradient; Aning, 2017; Pärtel et al., 2011; Ronk et al., 2015; Zobel, 1997), community completeness can provide a different aspect of biodiversity as it accounts for the variation in species pool size and expresses biodiversity on a relative scale (Pärtel et al., 2013). For instance, completeness exhibited no relationships to latitudinal gradients, but strong relations to anthropogenic disturbance (higher completeness in areas with lower disturbance) for fungi (Pärtel, Öpik, et al., 2017), plants (Ronk et al., 2015, 2016), and birds (Cam, Nichols, Sauer, Hines, & Flather, 2000). Comparing the environmental processes influencing these biodiversity measurements can provide valuable information for better prioritization of resources and understanding patterns of biodiversity. However, despite observed species richness and its determining factors being relatively established, dark diversity and its completeness counterpart are new methodologies, and as such, have not been well investigated, and the factors influencing them are not fully understood.

Determining the set of species that can theoretically inhabit a site, the species pool, is typically estimated using species co‐occurrence patterns with Beal's smoothing which assumes species with shared ecological requirements and biogeographic history will have similar likelihoods of being present at a particular site (Beals, 1984; de Bello et al., 2012; Lewis et al., 2016; McCune, 1994; Münzbergová & Herben, 2004). Since particular pairs of species will be less likely to co‐occur if they compete for the same resources, this approach also assumes to account for competitive interactions which are a major factor influencing species occurrence patterns (Cornell & Harrison, 2014; de Bello et al., 2012), especially in plant communities (Riibak et al., 2015). Although the Beals co‐occurrence matrix approach is expected to account for biotic interactions in the regional pool estimates, competitive exclusion can lead to lack of species at sites, thereby increasing dark diversity and decreasing species richness. To examine this further, we used Grime's plant life strategies (competitor/stress‐tolerator/ruderal, C‐S‐R (Grime, 1979)) to quantify the occurrence of competitive species in plant communities (Ejrnæs & Bruun, 2000); that is, when a site has a high Grime C value, it is dominated by few, but abundant, competitive species.

One way to consider the roles these factors play in dark diversity measurements can be provided with the recently developed ecospace framework (Brunbjerg et al., 2017). Ecospace divides the environmental causes of variation in species diversity into three main domains: (1) the position in environmental hyperspace (position), (2) the availability and variation of biotic resources (expansion), and (3) the spatiotemporal extent of habitats (continuity). This framework can be used to quantify and examine the roles of, for example, environmental filtering (position), as well as succession and human disturbance (continuity) on dark diversity. Ecospace also recognizes the role of vegetation structure and diversification of organic matter (expansion), as a contributing factor of biodiversity, bringing to light often‐ignored trophic interactions that exist between taxa (Brunbjerg et al., 2017). The importance of expansion on biodiversity has been illustrated by a recent study where vegetation structure was found to influence biodiversity across trophic groups of plants, animals, fungi, and bacteria (Penone et al., 2019). This framework unites theories such as niche theory, island biogeography theory, and a suite of community assembly theories into one framework for further development of a general theory of terrestrial biodiversity (Brunbjerg et al., 2017).

In this study, we investigate the importance of local environmental conditions and competition for dark diversity and completeness in plant communities across habitats and compare with results for observed plant richness. We ask what the relative importance of the ecospace dimensions is across habitats, and when habitat differences are accounted for in the diversity measure. We discuss how dark diversity can contribute to new aspects for informed conservation and management.

## 2 MATERIALS AND METHODS

### 2.1 Study sites

**FIGURE 1:**
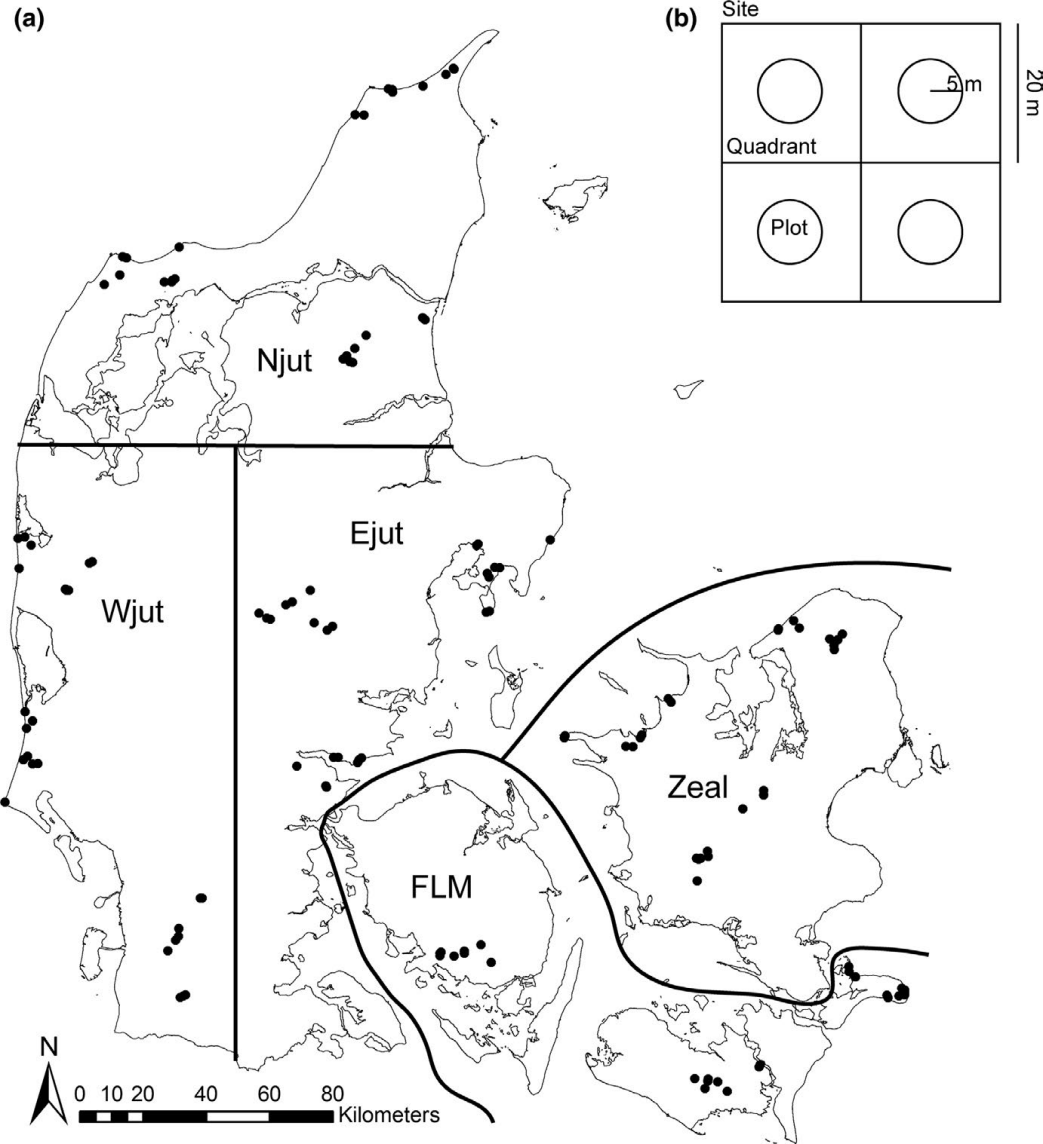
(a) Map of Denmark showing the 130 surveyed sites and the regions. (b) 40 m × 40 m site with the four quadrants and 5‐m circle plots. Reprinted and modified from Ejrnæs et al. (2018) copyright (2018), with permission from Elsevier

Our data stem from Biowide (www.biowide.dk), a nationwide survey of biodiversity in Denmark (Brunbjerg, Bruun, Broendum, et al., 2019). A total of 130 study sites (40 m × 40 m) were evenly distributed across five geographic regions in Denmark with a minimum distance of 500 m between sites (Figure 1a). Each site is sampled in four 5‐m circle plots (Figure 1b). Sampling was designed to evaluate the ecospace framework, stating that biodiversity varies according to abiotic conditions, buildup and diversification of organic resources, and spatiotemporal continuity (Brunbjerg et al., 2017). The sites were stratified to represent the main variation in major environmental gradients. Thirty sites were cultivated habitats and 100 sites natural and semi‐natural habitats. The cultivated subset was stratified to represent major land‐use types, and the natural subset was stratified to represent major gradients in soil fertility, soil moisture, and successional stage. Saline and fully aquatic habitats were deliberately excluded, but temporarily inundated depressions, as well as wet mires and fens, were included. The final set of 24 environmental strata consisted of the following six cultivated habitat types: three types of fields (rotational, grass leys, and set aside) and three types of plantations (beech, oak, and spruce). The other 18 strata covered natural and semi‐natural habitats, constituting all factorial combinations of fertile and infertile; dry, moist, and wet; and open, tall herb/scrub, and forest. All 24 strata were replicated in each of the five geographic regions. Intensively (ploughed and fertilized) managed agricultural fields differ markedly in the processes determining plant communities and were therefore excluded from this study resulting in a dataset of 115 sites. All fieldwork and sampling were conducted in accordance with Responsible Research at Aarhus University and Danish law. For a thorough description of site selection and stratification procedures, see Brunbjerg, Bruun, Broendum, et al. (2019).

### 2.2 Data

#### 2.2.1 Plant species richness

Vascular plant species richness was inventoried in 5‐m circular plots at each site (four plots at each site) by trained botanists during the summer 2014 and spring 2015 to account for variations in phenology. We removed all subspecies, hybrids, variations, and neophytes (i.e., species that are not considered a natural part of the vegetation given their history and dispersal ability; see appendix tables 6–8 in Buchwald et al., 2013). The nomenclature in this study follows the species checklist Denmark database from https://allearter‐databasen.dk. The data contained a total of 580 species of vascular plants in the 115 sites spanning from open habitats to shrubs and late‐succession forests.

#### 2.2.2 Explanatory variables

According to ecospace, the position variables included in the model were soil moisture index (SMI) and soil fertility index (SFI). For each site, SFI represents the predicted value from the best linear model (of all sites) of site mean Ellenberg N (plant‐based bioindication of nutrient status (Ellenberg et al., 1991)) as a function of soil Ca, leaf N, leaf NP, and soil type. We calculated a soil moisture index for each site using the predicted values from the best linear model (of all sites) of mean Ellenberg F (plant‐based bioindication of soil moisture; Ellenberg et al., 1991) as a function of mean precipitation in 2001–2010 (10 km × 10 km grid resolution) and measured site soil moisture (trimmed mean of 16 measures pr. site taken with a FieldScout TDR 300 Soil Moisture Meter in May 2016; Brunbjerg, Bruun, Dalby, et al., 2019). Position also included soil pH measured in four pooled soil samples from 0 to 10 cm depth and light measured as light intensity (Lux) using HOBO Pendant^®^ Temperature/Light 8K Data Loggers installed at the ground as detailed in Brunbjerg, Bruun, Broendum, et al. (2019). The expansion variables included were as follows: (a) bare soil percent coverage as an approximate estimate, (b) litter mass (g/m^2^ of four dried (60° for 48 hr) litter samples within a 21 cm × 21 cm frame pr. site), (c) soil organic matter as a percentage of the 0–10 cm soil core that was categorized as organic soil, (d) soil organic carbon as % soil C in 0–10 cm soil layer (g/m^2^ average of four soil samples taken at each site) as described in Brunbjerg, Bruun, Broendum, et al. (2019), and (e) vegetation heterogeneity derived from Lidar. The latter included both canopy height variation (variance of the 90th percentile for points >3 m within the site) and shrub layer height variation (variance of the 90th percentile for returns between 30 cm and 3 m [Brunbjerg, Bruun, Dalby, et al., 2019]). Spatial continuity included landscape characteristics: (a) fraction of intensive fields within a 500‐m buffer of the site and (b) the fraction of natural habitats in a 1 km × 1 km grid from a national mapping, interpolated using Spline in ArcGIS 10.2.2 (weight 0.5, number of points 9 [Ejrnæs et al., 2014]). Temporal continuity was estimated directly for each study site as time since major land‐use change: For each site, a temporal sequence of aerial photographs and historical maps was inspected starting with the most recent photographs (photographs from 2014, 2012, 2010, 2008, 2006, 2004, 2002, 1999, 1995, 1968, 1954, 1945) and ending with historical maps reflecting land use in the period 1842–1945. Temporal continuity (the year in which a change could be identified) was reclassified into a numeric 4‐level variable: 1: 1–14 years, 2: 15–44 years, 3: 45–135 years, and 4: >135 years (Brunbjerg, Bruun, Broendum, et al., 2019).

Lastly, to examine the assumption that competitive exclusion can lead to lack of co‐occurrence and hence increased dark diversity, we used Grime's plant life strategies to quantify the importance of local interspecific competition for establishment of species. Grime's plant strategy theory (competitor/stress‐tolerator/ruderal, C‐S‐R) states a three‐way trade‐off between life strategies that facilitate competition for resources (competitive strategy), survival in stressful environments, for example, with high salinity, flooding, and drought (stress tolerance), and survival in disturbed environments (ruderalism; Grime, 1979). The original C‐S‐R species strategies were recoded as numeric values for each plant species where a total of 12 points were distributed to the different strategies as described in Ejrnæs and Bruun (2000). To represent competitive ability, we used the unweighted mean site *C* value based on the site species lists.

### 2.3 Data analysis

#### 2.3.1 Regional pool, dark diversity, and completeness

All statistical analyses were performed in R version 3.5.3 (R Core Team, 2019). To calculate regional pools, we used vegetation data from the 5‐m circular Biowide plots (four at each site) as well as an additional dataset of plant inventories in 5‐m circles from the national monitoring program (Danish Nature Agency, 2016). This dataset includes 52,362 plots with more than five species recorded by trained botanists and is added to increase the co‐occurrence matrix for robustness in the Beal's calculations of regional pools (see below). We did not include species‐poor plots, that is, those with less than five observed species, resulting in 448 plots from Biowide and 52,362 plots from the additional dataset. The regional pool was calculated using the Beals index (Beals, 1984), as recommended by Lewis et al. (2016). The Beals index represents the probability that a particular species will occur within a given site based on the assemblage of co‐occurring species (Beals, 1984; McCune, 1994; Münzbergová & Herben, 2004). We calculated the Beals index using the “beals” function in the “vegan” package (Oksanen et al., 2017). The threshold for including a particular species in the regional species pool is recommended to be the 5th percentile of the Beals index value for the species in question (Gijbels, Adriaens, & Honnay, 2012; Ronk et al., 2015). Preceding the calculation of each threshold, the lowest Beals index value among plots with occurrence of the species in question was identified, and all plots having values below that minimum were not considered.

**FIGURE 2:**
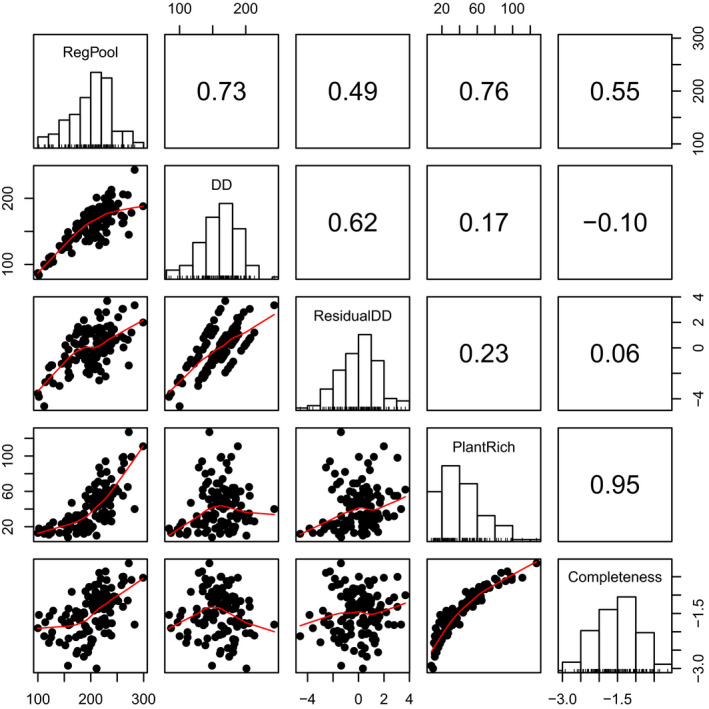
Spearman rank correlations and dot plots of site regional pool, dark diversity, residual dark diversity, plant species richness, and completeness. The red line in the plot shows a loess smoothing line

Analyses were done at the site level (*n* = 115) by creating a site regional pool combining the four plot regional pools at each site. In addition to the four plots, plants had been inventories in the whole site, and therefore observed species in the site, but not included in the regional pools (*n* = 2) were added to the regional pools to ensure that site regional pool included all observed species. Then, dark diversity was calculated for each site as the difference between the regional pool and the observed species richness (Pärtel et al., 2011) and completeness was calculated following Pärtel et al. (2013) using the formula *ln(observed richness/dark diversity)*. Because dark diversity is relative it may not be suitable for comparison across habitats (Scott, Alofs, & Edwards, 2011), and completeness is suggested as a possible alternative (Pärtel et al., 2013). In this study, we found that completeness was highly correlated with observed species richness (Figure 2), so instead, we propose a dark diversity measure corrected for habitat types using the residuals of a model of dark diversity as a function of the habitat type. To represent habitat type, we conducted a supervised classification of nine a priori defined training classes and using ordination gradients from an NMDS ordination based on all identified species, that is, plants, fungi, and insects from the Biowide project. The classification resulted in eight classes spanning gradients in succession (early, late), moisture (wet, dry), and nutrients (rich, poor). The habitat types explained 51% of the variation in dark diversity.

#### 2.3.2 Statistical analyses

Soil pH, litter mass, organic carbon, organic matter, and vegetation variation were log‐transformed, and all explanatory variables were standardized. We conducted model selection by first testing for multicollinearity using the variance inflation factor (VIF; Zuur, Ieno, & Elphick, 2010). We removed canopy height variation and organic matter resulting in a maximum VIF of 2.9. The remaining variables were used as explanatory variables (linear terms) in a set of generalized linear models (GLMs) with Poisson distribution for the count data for dark diversity and plant richness, and normal distribution for residual dark diversity, but as models for plant richness were overdispersed, we chose negative binomial models for this response instead. To avoid spurious correlation in the models, we excluded variables with no hypothesized relationship and constrained the response direction and shape to ecologically plausible responses (Burnham & Anderson, 2002; Zuur et al., 2010). In general, more resources, more diverse resources, more environmental variation, and increasing temporal and spatial continuity are all hypothesized to increase plant species richness. Exceptions to this are litter mass, intensive fields, and competitive ability, which are expected to decrease plant species richness. Generally, we expect the opposite relationships with dark diversity. Following this approach, we excluded negative effects of expansion, continuity, and heterogeneity variables on plant species richness. We did not include interaction terms because of lacking plausible ecological hypotheses. To allow for nonlinear relationships for position variables corresponding to the intermediate disturbance hypothesis (Connell, 1978; Townsend, Scarsbrook, & Dolédec, 1997) and intermediate productivity hypothesis (Fraser et al., 2015), we used AIC (Burnham & Anderson, 2002) to evaluate whether inclusion of quadratic terms for the variables SMI, SFI, light, soil pH, and bare soil improved the model fit (using delta AIC < 2 as a rule), in which case relevant quadratic terms were added. Subsequently, we checked again the responses’ direction and excluded ecologically implausible responses (Burnham & Anderson, 2002). We then dropped remaining variables sequentially based on AIC using the function drop1() in the lme4 package (Bates, Sarkar, Bates, & Matrix, 2007). The final regression models were tested for overdispersion and evaluated by visual inspection of residual plots and for spatial autocorrelation using Moran's I in the ape package (Paradis, Claude, & Strimmer, 2004).

## 3 RESULTS

**FIGURE 3:**
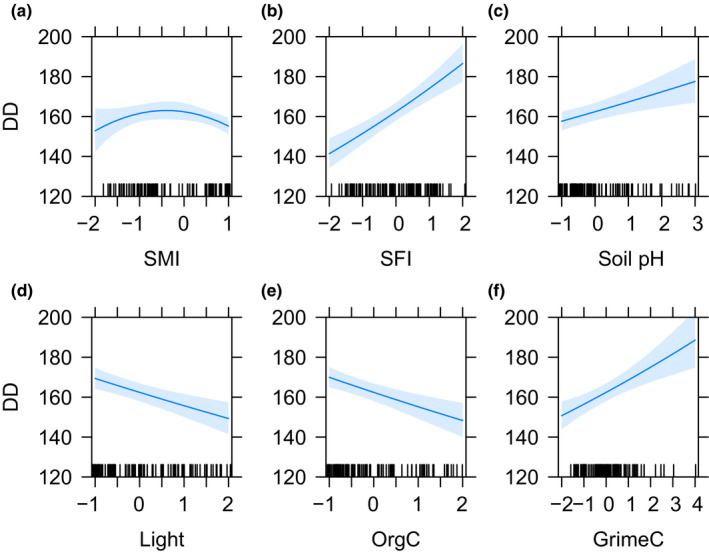
Parameter estimates with 95% confidence intervals from the significant environmental variables predicting overall dark diversity. Relationships between the dark diversity and (a) soil moisture index (SMI), (b) soil fertility index, (c) soil pH, (d) light, (e) organic matter, and (f) plants’ competitive ability (GrimeC). The *y*‐axis is truncated

**FIGURE 4:**
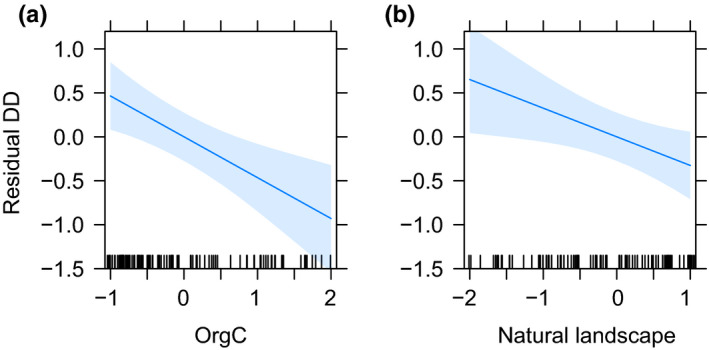
Parameter estimates with 95% confidence intervals from the significant environmental variables predicting residual dark diversity for habitat types. Relationships between residual dark diversity and (a) organic carbon (OrgC), and (b) fraction of natural landscape

**FIGURE 5:**
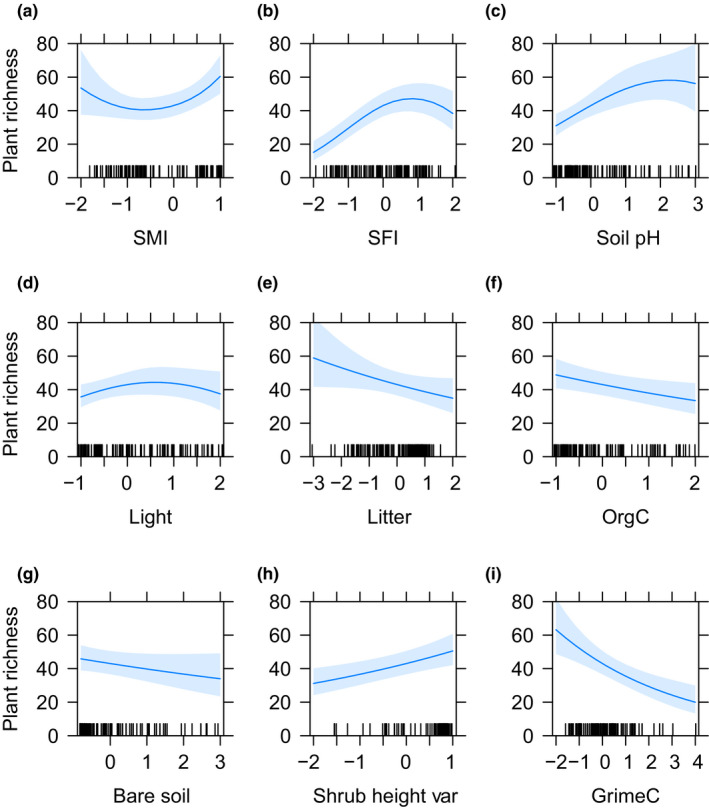
Parameter estimates with 95% confidence intervals from the significant environmental variables predicting plant species richness. Relationships between the plant species richness and (a) soil moisture index (SMI), (b) soil fertility index (SFI) (c) soil pH, (d) litter mass, (f) organic carbon, (g) bare soil, (h) shrub height variation, and (i) plants’ competitive ability (GrimeC)

The species richness per site ranged from 8 to 127 species, and dark diversity ranged from 84 to 243 species. Completeness and plant species richness were highly positively correlated (*r*_s_ = .95, Figure 2), and completeness was therefore excluded from further analysis. Dark diversity was less correlated with plant species richness (*r*_s_ = .17, Figure 2). The final regression models explained between 14% and 65% of the variation in dark diversity, residual dark diversity, and species richness (Table 1). We found position variables to be important for dark diversity and plant species richness (Figures 3 and 5). Soil moisture invoked a unimodal response in dark diversity but a bimodal response in species richness with a low at intermediate soil moisture. We observed a positive effect of soil fertility and soil pH on dark diversity and unimodal relationships with species richness with peaks at intermediate to high fertility and soil pH. Light had a negative linear relationship with dark diversity and a unimodal relationship with species richness with a peak in richness at intermediate light conditions. No position variables were found to be important for the residual dark diversity, as would be expected. Organic carbon was important for all measured responses with a linear negative relationship across response variables (Figures 3, 4, 5). Community mean competitive ability (Grime C) had a positive linear relationship with dark diversity and a negative linear relationship with plant species richness (Figures 3 and 5), while natural landscapes had a linear negative relationship with residual dark diversity (Figure 4).

**TABLE 1:** Regression models of dark diversity (DD), residual dark diversity (Residual DD), and plant species richness (PlantRich)

Ecospace	Variables	DD	Residual DD	Plant richness
	Intercept	5.090*** (0.014)	−0.000 (0.136)	3.762*** (0.081)
Position	Soil moisture index (SMI)	−0.020* (0.011)	n.s.	0.190*** (0.054)
Position	SMI^2^	−0.025** (0.012)	n.s.	0.150*** (0.053)
Position	Soil fertility index (SFI)	0.069*** (0.011)	n.s.	0.233*** (0.055)
Position	SFI^2^	n.s.	n.s.	−0.145*** (0.029)
Position	Soil pH	0.030*** (0.009)	n.s.	0.269*** (0.071)
Position	Soil pH^2^	n.s.	n.s.	−0.060* (0.034)
Position	Light	−0.042*** (0.010)	n.s.	0.102 (0.066)
Position	Light^2^	n.s.	n.s.	−0.086* (0.047)
Expansion	Litter (log)	n.s.	n.s.	−0.105* (0.056)
Expansion	Organic carbon (OrgC)	−0.045*** (0.011)	−0.465*** (0.137)	−0.126** (0.050)
Expansion	Bare soil	n.s.	n.s.	−0.079 (0.050)
Expansion	Shrub height variation	n.s.	n.s.	0.161*** (0.048)
Interaction	Competition (Grime C)	0.037*** (0.009)	n.s.	−0.192*** (0.048)
Continuity	Natural Landscape	n.s.	−0.326** (0.137)	n.s.
	Multiple *R* ^2^	.65	.14	.65

Note

Explanatory variables are framed according to ecospace position, expansion, and continuity with the addition of competition. We use ordinary least squares with Poisson distribution for DD and negative binomial for PlantRich. *R* ^2^ is calculated as 1 − (model deviance/model null deviance) for dark diversity and plant species richness. For residual dark diversity (DD), we report the multiple *R* ^2^. Standard errors are given in parentheses.

* *p* < .1.

** *p* < .05.

*** *p* < .01.

## 4 DISCUSSION

In this study, we found position variables to be important for both plant richness and dark diversity. Once abiotic and successional differences between habitat types were accounted for, that is, residual dark diversity, the position variables were no longer significant indicating that the habitat classification used here is largely driven by abiotic gradients. Plant richness was highest at intermediate conditions of soil fertility and pH, corresponding to the intermediate productivity hypothesis, which states that few species can tolerate the environmental stresses at low productivity and a few highly competitive species dominate at high productivity (Fraser et al., 2015). Species richness increased with pH, possibly corresponding to the generally large regional species pool in calcareous habitats, and aligns with previous research indicating that plant diversity has a strong positive association with soil pH in temperate and boreal regions (Pärtel, 2002; Pärtel, Helm, Ingerpuu, Reier, & Tuvi, 2004). The unimodal relationship between dark diversity and soil moisture may reflect that hydrological gradients can be strong environmental filters determining plant communities (Fraaije et al., 2015; Silvertown, Araya, & Gowing, 2015; Valdez, Hartig, Fennel, & Poschlod, 2019) with more distinct communities at the extremes than at intermediate soil moisture, that is, specific adaptations for waterlogged and very dry soil are required (Ernst, 1990). Therefore, fewer coincidental species may appear in these extreme habitats compared with habitats of intermediate soil moisture, resulting in a low estimated regional pool and lower dark diversity at the extremes.

Expansion of ecospace seems more important for plant species richness and less important for dark diversity as only organic carbon is significant for dark diversity. This is somewhat unexpected as many of the hypotheses for associations between expansion and richness would also apply inversely to dark diversity. For example, variation in shrub height was positively correlated with species richness corresponding to a general positive effect of vegetation heterogeneity on species richness by increasing available niche space and providing grazing refuges (Stein, Gerstner, & Kreft, 2014). In our experience, shrub height variation also reflects land‐use history, that is, intensive, mechanical land use causes homogenization and general low vegetation that concurs with low species richness. Concordantly, shrub height variation should be negatively related to dark diversity; however, it is insignificant. Species richness was negatively related to litter mass corresponding to a general negative effect of litter on germination, establishment, richness, and biomass of plants (Xiong & Nilsson, 1999). This effect, however, does not seem to apply to dark diversity, where litter mass is insignificant. We found a negative relationship between bare soil and species richness, indicating that although bare soil is expected to increase the survival at germination (e.g., Roth, Seeger, Poschlod, Pfadenhauer, & Succow, 1999) and thereby affect community composition and increase plant richness, it may also reflect a trade‐off where bare soil means fewer growing individuals and species. The only expansion variable to influence dark diversity, residual dark diversity, and species richness was soil organic carbon, influencing all three diversity measures negatively. While organic carbon may not be a biotic resource for plants in accordance with ecospace, it is related to long‐term stability in late‐successional stages as soil organic carbon accumulates over time (Luyssaert et al., 2008). While long‐term stability and hence time for establishment imply lower dark diversity, high organic carbon also coincides with species‐poor habitats, such as forests, mires, bogs, and heaths.

For the first time, we attempted to investigate the effect of competition on dark diversity. The importance of competition for dark diversity hints that the regional pool estimate using Beals does not fully account for biotic interactions although it is expected from the co‐occurrence approach inherent in the Beals index (Cornell & Harrison, 2014; de Bello et al., 2012; Riibak et al., 2015). However, scale may also play a role. The plant communities used in this study cover broad environmental gradients (including the additional dataset of 52,362 plots), and when estimating the regional pool from the co‐occurrence matrix, abiotic filtering may therefore be more prevalent over biotic filtering (Kraft et al., 2015). High competitive exclusion as a biotic filtering of the regional pool may still occur at sites with relatively few but abundant, competitive species and implies that other species are missing. This corresponds to our findings of both decreased species richness and increased dark diversity in communities characterized by competitive species (high community mean GrimeC). Excluded species from the regional pool can colonize where communities dominated by competitive species are disturbed (e.g., by grazing, erosion, or other). On the contrary, competition was not significant for the residual dark diversity. It is possible that accounting for the variation in habitat types not only accounts for variation in position but also the inherent competitive strategies of the species in the habitat types, for example, late‐successional stages with long‐term stability are generally dominated by competitive species. Recent review and opinion papers (Cadotte & Tucker, 2017; Kraft et al., 2015) have highlighted that biotic interactions of species may be related to environmental gradients. Residual dark diversity seems to decrease with the fraction of natural land in the surroundings of the sites possibly by influencing local processes, that is, landscapes with high density of nature are likely to have a higher local pool of species, increased dispersal, increased species survival, metapopulation structures, and less negative edge effects of intensive land use (Brunbjerg et al., 2017). It is possible that this effect is otherwise masked by the large effect of habitat type and position variables in both dark diversity and observed diversity, making the effect of natural landscapes only visible once this other variation has been accounted for.

This study also shows that different diversity measures contribute with different aspects to better understand drivers of diversity. For example, the negative effect of organic carbon on plant species richness could indicate that carbon storage leads to loss of species richness, whereas the effect on dark diversity actually indicates that fewer species are missing when carbon storage is high. Here, we compared dark diversity and completeness, thought to be less dependent on habitat types (Pärtel et al., 2013). However, we found that completeness was highly correlated with observed species richness and therefore added no new information to our study of plant community diversity aspects. We therefore corrected for differences in regional pool through a residual dark diversity to provide a measure comparable across habitats. Comparing the models of dark diversity and residual dark diversity, similar results are obtained by including major position variables as dependent variables in the model for dark diversity. When examining dark diversity, we recommend to compare and discuss the results of different diversity measures.

With global biodiversity rapidly decreasing, it is vital to understand the drivers of biodiversity to prioritize conservation and make management more efficient. In this study, besides the ecospace position variables, competition seems to be the strongest predictor of plant richness. Conservation management focusing on intermediate disturbance such as grazing can disturb competitive communities making room for more species and thereby decrease dark diversity. Besides ecospace position, organic carbon was the most important variable for both dark diversity measures indicating that long‐term stability in late‐successional habitats decreases dark diversity. Examining the influencing factors of different measures of biodiversity can lead to better decision‐making in the future conservation of the world's biodiversity.

## CONFLICT OF INTEREST

None declared.

## AUTHOR CONTRIBUTION

**Camilla Fløjgaard:** Conceptualization (equal); Formal analysis (lead); Methodology (lead); Validation (equal); Visualization (equal); Writing‐original draft (equal); Writing‐review & editing (lead). **José W. Valdez:** Writing‐original draft (lead); Writing‐review & editing (equal). **Lars Dalby:** Conceptualization (equal); Data curation (lead); Formal analysis (equal); Methodology (equal); Validation (equal); Writing‐original draft (supporting); Writing‐review & editing (supporting). **Jesper Erenskjold Moeslund:** Conceptualization (equal); Data curation (lead); Formal analysis (supporting); Funding acquisition (lead); Methodology (equal); Project administration (lead); Validation (supporting); Writing‐original draft (supporting); Writing‐review & editing (supporting). **Kevin K. Clausen:** Conceptualization (equal); Formal analysis (supporting); Methodology (supporting); Writing‐original draft (supporting); Writing‐review & editing (supporting). **Rasmus Ejrnæs:** Conceptualization (equal); Data curation (equal); Methodology (supporting); Writing‐original draft (supporting); Writing‐review & editing (supporting). **Meelis Partel:** Conceptualization (supporting); Formal analysis (supporting); Methodology (equal). **Ane Kirstine Brunbjerg:** Conceptualization (equal); Data curation (equal); Formal analysis (lead); Methodology (equal); Validation (lead); Visualization (lead); Writing‐original draft (equal); Writing‐review & editing (equal).

## Data Availability

Plant species lists from 5‐m plots and environmental variables: Dryad https://doi.org/10.5061/dryad.9kd51c5d5.

## References

[ece36351-bib-0001] Aning, J. V. (2017). Diversity and completeness of North American mammal assemblages. M.Sc., Utrecht University.

[ece36351-bib-0002] Bartelheimer, M. , & Poschlod, P. (2016). Functional characterizations of Ellenberg indicator values–a review on ecophysiological determinants. Functional Ecology, 30, 506–516.

[ece36351-bib-0003] Bates, D. , Sarkar, D. , Bates, M. D. , & Matrix, L. (2007). The lme4 package. R package version, 2, 74.

[ece36351-bib-0004] Beals, E. W. (1984). Bray‐Curtis ordination: An effective strategy for analysis of multivariate ecological data In MacFadyen A., & Ford E. D. (Eds.), Advances in ecological research. Cambridge, MA: Academic Press.

[ece36351-bib-0005] Bello, F. , Fibich, P. , Zelený, D. , Kopecký, M. , Mudrák, O. , Chytrý, M. , … Pärtel, M. (2016). Measuring size and composition of species pools: A comparison of dark diversity estimates. Ecology and Evolution, 6, 4088–4101. 10.1002/ece3.2169 27516866 PMC4877358

[ece36351-bib-0006] Bennett, J. A. , Riibak, K. , Kook, E. , Reier, Ü. , Tamme, R. , Guillermo Bueno, C. , & Pärtel, M. (2016). Species pools, community completeness and invasion: Disentangling diversity effects on the establishment of native and alien species. Ecology Letters, 19, 1496–1505. 10.1111/ele.12702 27882703

[ece36351-bib-0007] Boussarie, G. , Bakker, J. , Wangensteen, O. S. , Mariani, S. , Bonnin, L. , Juhel, J.‐B. , … Mouillot, D. (2018). Environmental DNA illuminates the dark diversity of sharks. Science Advances, 4, eaap9661 10.1126/sciadv.aap9661 29732403 PMC5931749

[ece36351-bib-0008] Brunbjerg, A. K. , Bruun, H. H. , Broendum, L. , Classen, A. T. , Fog, K. , Froeslev, T. G. , … Ejrnaes, R. (2019). A systematic survey of regional multitaxon biodiversity: Evaluating strategies and coverage. BMC Ecology, 19, 158030 10.1101/158030 PMC6792264 31615504

[ece36351-bib-0009] Brunbjerg, A. K. , Bruun, H. H. , Dalby, L. , Classen, A. T. , Fløjgaard, C. , Frøslev, T. G. , … Ejrnæs, R. (2019). Multitaxon inventory reveals highly consistent biodiversity responses to ecospace variation. BioRxiv, 807321, 10.1101/807321

[ece36351-bib-0010] Brunbjerg, A. K. , Bruun, H. H. , Dalby, L. , Fløjgaard, C. , Frøslev, T. G. , Høye, T. T. , … Ejrnæs, R. (2018). Vascular plant species richness and bioindication predict multi‐taxon species richness. Methods in Ecology and Evolution, 9, 2372–2382. 10.1111/2041-210X.13087

[ece36351-bib-0011] Brunbjerg, A. K. , Bruun, H. H. , Moeslund, J. E. , Sadler, J. P. , Svenning, J.‐C. , & Ejrnæs, R. (2017). Ecospace: A unified framework for understanding variation in terrestrial biodiversity. Basic and Applied Ecology, 18, 86–94. 10.1016/j.baae.2016.09.002

[ece36351-bib-0012] Buchwald, E. , Wind, P. , Bruun, H. H. , Møller, P. F. , Ejrnæs, R. , & Svart, H. E. (2013). Hvilke planter er hjemmehørende i Danmark? Flora & Fauna, 118, 73–96.

[ece36351-bib-0013] Burnham, K. P. , & Anderson, D. R. (2002). Model selection and multi‐model inference: A practical information‐theoretic approach. New York, NY: Springer.

[ece36351-bib-0014] Butchart, S. H. M. , Walpole, M. , Collen, B. , van Strien, A. , Scharlemann, J. P. W. , Almond, R. E. A. , … Watson, R. (2010). Global biodiversity: Indicators of recent declines. Science, 328, 1164–1168. 10.1126/science.1187512 20430971

[ece36351-bib-0015] Cadotte, M. W. , & Tucker, C. M. (2017). Should environmental filtering be abandoned? Trends in Ecology & Evolution, 32, 429–437. 10.1016/j.tree.2017.03.004 28363350

[ece36351-bib-0016] Cam, E. , Nichols, J. D. , Sauer, J. R. , Hines, J. E. , & Flather, C. H. (2000). Relative species richness and community completeness: Birds and urbanization in the mid‐Atlantic states. Ecological Applications, 10, 1196–1210. 10.1890/1051-0761(2000)010[1196:RSRACC]2.0.CO;2

[ece36351-bib-0017] Connell, J. H. (1978). Diversity in tropical rain forests and coral reefs. Science, 199, 1302–1310. 10.1126/science.199.4335.1302 17840770

[ece36351-bib-0018] Cornell, H. V. , & Harrison, S. P. (2014). What are species pools and when are they important? Annual Review of Ecology, Evolution, and Systematics, 45, 45–67. 10.1146/annurev-ecolsys-120213-091759

[ece36351-bib-0019] Danish Nature Agency (2016). Vascular plants in Denmark recorded under the The Nationwide Monitoring and Assessment Programme for the Aquatic and Terrestrial Environments (NOVANA). Occurrence Dataset. 9.1 ed. Global Biodiversity Information Facility.

[ece36351-bib-0020] de Bello, F. , Price, J. N. , Münkemüller, T. , Liira, J. , Zobel, M. , Thuiller, W. , … Pärtel, M. (2012). Functional species pool framework to test for biotic effects on community assembly. Ecology, 93, 2263–2273. 10.1890/11-1394.1 23185887

[ece36351-bib-0021] Deangelis, D. L. (2012). Dynamics of nutrient cycling and food webs. Berlin, Germany: Springer Science & Business Media.

[ece36351-bib-0022] Díaz, S. , Settele, J. , Brondízio, E. , Ngo, H. T. , Guèze, M. , Agard, J. , & Zayas, C. (2019). Summary for policymakers of the global assessment report on biodiversity and ecosystem services of the Intergovernmental Science‐Policy Platform on Biodiversity and Ecosystem Services InCarneiro da Cunha M., Mace G., & Mooney H. (Eds.), IPBES‐7th Plenary, 6 May 2019, Paris, France. Intergovernmental Science‐Policy Platform on Biodiversity and Ecosystem Services.

[ece36351-bib-0023] Ejrnæs, R. , & Bruun, H. H. (2000). Gradient analysis of dry grassland vegetation in Denmark. Journal of Vegetation Science, 11, 573–584. 10.2307/3246587

[ece36351-bib-0024] Ejrnæs, R. , Frøslev, T. G. , Høye, T. T. , Kjøller, R. , Oddershede, A. , Brunbjerg, A. K. , … Bruun, H. H. (2018). Uniquity: A general metric for biotic uniqueness of sites. Biological Conservation, 225, 98–105. 10.1016/j.biocon.2018.06.034

[ece36351-bib-0025] Ejrnæs, R. , Petersen, A. H. , Bladt, J. , Bruun, H. H. , Moeslund, J. E. , Wiberg‐Larsen, P. , & Rahbek, C. (2014). Biodiversitetskort for Danmark: Udviklet i samarbejde mellem Center for Makroøkologi, Evolution og Klima på Københavns Universitet og Institut for Bioscience ved Aarhus Universitet nr. 112, Aarhus Universitet, DCE‐Nationalt Center for Miljø og Energi.

[ece36351-bib-0026] Ellenberg, H. , Weber, H. E. , Düll, R. , Wirth, V. , Werner, W. , & Paulißen, D. (1991). Zeigerwerte von pflanzen in Mitteleuropa. Scripta Geobotanica, 18, 1–248.

[ece36351-bib-0027] Ernst, W. H. O. (1990). Ecophysiology of plants in waterlogged and flooded environments. Aquatic Botany, 38, 73–90.

[ece36351-bib-0028] Estrada, A. , Márcia Barbosa, A. , & Real, R. (2018). Changes in potential mammal diversity in National Parks and their implications for conservation. Current Zoology, 64, 671–679. 10.1093/cz/zoy001 30538726 PMC6280100

[ece36351-bib-0029] Fraaije, R. G. , Ter Braak, C. J. , Verduyn, B. , Breeman, L. B. , Verhoeven, J. T. , & Soons, M. B. (2015). Early plant recruitment stages set the template for the development of vegetation patterns along a hydrological gradient. Functional Ecology, 29, 971–980. 10.1111/1365-2435.12441

[ece36351-bib-0030] Fraser, L. H. , Pither, J. , Jentsch, A. , Sternberg, M. , Zobel, M. , Askarizadeh, D. , … Zupo, T. (2015). Worldwide evidence of a unimodal relationship between productivity and plant species richness. Science, 349, 302–305. 10.1126/science.aab3916 26185249

[ece36351-bib-0031] Gijbels, P. , Adriaens, D. , & Honnay, O. (2012). An orchid colonization credit in restored calcareous grasslands. Ecoscience, 19, 21–28. 10.2980/19-1-3460

[ece36351-bib-0032] Grime, J. P. (1979). Plant strategies and vegetation processes. Chichester, UK: Wiley.

[ece36351-bib-0033] Kraft, N. J. , Adler, P. B. , Godoy, O. , James, E. C. , Fuller, S. , & Levine, J. M. (2015). Community assembly, coexistence and the environmental filtering metaphor. Functional Ecology, 29, 592–599. 10.1111/1365-2435.12345

[ece36351-bib-0034] Lewis, R. J. , Szava‐Kovats, R. , & Pärtel, M. (2016). Estimating dark diversity and species pools: An empirical assessment of two methods. Methods in Ecology and Evolution, 7, 104–113. 10.1111/2041-210X.12443

[ece36351-bib-0035] Luyssaert, S. , Schulze, E. D. , Borner, A. , Knohl, A. , Hessenmoller, D. , Law, B. E. , … Grace, J. (2008). Old‐growth forests as global carbon sinks. Nature, 455, 213–215. 10.1038/nature07276 18784722

[ece36351-bib-0036] Mccune, B. (1994). Improving community analysis with the Beals smoothing function. Ecoscience, 1, 82–86.

[ece36351-bib-0037] Moeslund, J. E. , Brunbjerg, A. K. , Clausen, K. K. , Dalby, L. , Fløjgaard, C. , Juel, A. , & Lenoir, J. (2017). Using dark diversity and plant characteristics to guide conservation and restoration. Journal of Applied Ecology, 54, 1730–1741. 10.1111/1365-2664.12867

[ece36351-bib-0038] Münzbergová, Z. , & Herben, T. (2004). Identification of suitable unoccupied habitats in metapopulation studies using co‐occurrence of species. Oikos, 105, 408–414. 10.1111/j.0030-1299.2004.13017.x

[ece36351-bib-0039] Oksanen, J. , Blanchet, F. G. , Kindt, R. , Legendre, P. , Minchin, P. R. , O'hara, R. , … Wagner, H. (2017). vegan: Community Ecology Package. R package version 2.4‐3.

[ece36351-bib-0040] Paradis, E. , Claude, J. , & Strimmer, K. (2004). APE: Analyses of phylogenetics and evolution in R language. Bioinformatics, 20, 289–290. 10.1093/bioinformatics/btg412 14734327

[ece36351-bib-0041] Pärtel, M. (2002). Local plant diversity patterns and evolutionary history at the regional scale. Ecology, 83, 2361–2366. 10.2307/3071796

[ece36351-bib-0042] Pärtel, M. (2014). Community ecology of absent species: Hidden and dark diversity. Journal of Vegetation Science, 25, 1154–1159. 10.1111/jvs.12169

[ece36351-bib-0043] Pärtel, M. , Helm, A. , Ingerpuu, N. , Reier, Ü. , & Tuvi, E.‐L. (2004). Conservation of northern European plant diversity: The correspondence with soil pH. Biological Conservation, 120, 525–531. 10.1016/j.biocon.2004.03.025

[ece36351-bib-0044] Pärtel, M. , Öpik, M. , Moora, M. , Tedersoo, L. , Szava‐Kovats, R. , Rosendahl, S. , … Zobel, M. (2017). Historical biome distribution and recent human disturbance shape the diversity of arbuscular mycorrhizal fungi. New Phytologist, 216, 227–238. 10.1111/nph.14695 28722181

[ece36351-bib-0045] Pärtel, M. , Szava‐Kovats, R. , & Zobel, M. (2011). Dark diversity: Shedding light on absent species. Trends in Ecology & Evolution, 26, 124–128. 10.1016/j.tree.2010.12.004 21195505

[ece36351-bib-0046] Pärtel, M. , Szava‐Kovats, R. , & Zobel, M. (2013). Community completeness: Linking local and dark diversity within the species pool concept. Folia Geobotanica, 48, 307–317. 10.1007/s12224-013-9169-x

[ece36351-bib-0047] Pärtel, M. , Zobel, M. , Öpik, M. , & Tedersoo, L. (2017). Global patterns in local and dark diversity, species pool size and community completeness in ectomycorrhizal fungi In Tedersoo L. (Ed.), Biogeography of Mycorrhizal Symbiosis. Cham, Switzerland: Springer International Publishing.

[ece36351-bib-0048] Pärtel, M. , Zobel, M. , Zobel, K. , van der Maarel, E. , & Partel, M. (1996). The species pool and its relation to species richness: Evidence from Estonian plant communities. Oikos, 75, 111–117. 10.2307/3546327

[ece36351-bib-0049] Penone, C. , Allan, E. , Soliveres, S. , Felipe‐Lucia, M. R. , Gossner, M. M. , Seibold, S. , … Fischer, M. (2019). Specialisation and diversity of multiple trophic groups are promoted by different forest features. Ecology Letters, 22, 170–180. 10.1111/ele.13182 30463104

[ece36351-bib-0050] R Core Team (2019). R: A language and environment for statistical computing 3.6.0 ed. Vienna, Austria: R Foundation for Statistical Computing Retrieved from https://www.R‐project.org/

[ece36351-bib-0051] Riibak, K. , Reitalu, T. , Tamme, R. , Helm, A. , Gerhold, P. , Znamenskiy, S. , … Pärtel, M. (2015). Dark diversity in dry calcareous grasslands is determined by dispersal ability and stress‐tolerance. Ecography, 38, 713–721. 10.1111/ecog.01312

[ece36351-bib-0052] Riibak, K. , Ronk, A. , Kattge, J. , & Pärtel, M. (2017). Dispersal limitation determines large‐scale dark diversity in Central and Northern Europe. Journal of Biogeography, 44, 1770–1780. 10.1111/jbi.13000

[ece36351-bib-0053] Ronk, A. , De Bello, F. , Fibich, P. , & Pärtel, M. (2016). Large‐scale dark diversity estimates: New perspectives with combined methods. Ecology and Evolution, 6, 6266–6281. 10.1002/ece3.2371 27648241 PMC5016647

[ece36351-bib-0054] Ronk, A. , Szava‐Kovats, R. , & Pärtel, M. (2015). Applying the dark diversity concept to plants at the European scale. Ecography, 38, 1015–1025. 10.1111/ecog.01236

[ece36351-bib-0055] Roth, S. , Seeger, T. , Poschlod, P. , Pfadenhauer, J. , & Succow, M. (1999). Establishment of helophytes in the course of fen restoration. Applied Vegetation Science, 2, 131–136. 10.2307/1478890

[ece36351-bib-0056] Scott, C. E. , Alofs, K. M. , & Edwards, B. A. (2011). Putting dark diversity in the spotlight. Trends in Ecology & Evolution, 26, 263–264. 10.1016/j.tree.2011.03.008 21470707

[ece36351-bib-0057] Silvertown, J. , Araya, Y. , & Gowing, D. (2015). Hydrological niches in terrestrial plant communities: A review. Journal of Ecology, 103, 93–108. 10.1111/1365-2745.12332

[ece36351-bib-0058] Stein, A. , Gerstner, K. , & Kreft, H. (2014). Environmental heterogeneity as a universal driver of species richness across taxa, biomes and spatial scales. Ecology Letters, 17, 866–880. 10.1111/ele.12277 24751205

[ece36351-bib-0059] Tittensor, D. P. , Walpole, M. , Hill, S. L. L. , Boyce, D. G. , Britten, G. L. , Burgess, N. D. , … Ye, Y. (2014). A mid‐term analysis of progress toward international biodiversity targets. Science, 346, 241–244. 10.1126/science.1257484 25278504

[ece36351-bib-0060] Townsend, C. R. , Scarsbrook, M. R. , & Dolédec, S. (1997). The intermediate disturbance hypothesis, refugia, and biodiversity in streams. Limnology and Oceanography, 42, 938–949. 10.4319/lo.1997.42.5.0938

[ece36351-bib-0061] Valdez, J. W. , Hartig, F. , Fennel, S. , & Poschlod, P. (2019). The recruitment niche predicts plant community assembly across a hydrological gradient along plowed and undisturbed transects in a former agricultural wetland. Frontiers in Plant Science, 10, 88 10.3389/fpls.2019.00088 30787938 PMC6372561

[ece36351-bib-0062] Xiong, S. , & Nilsson, C. (1999). The effects of plant litter on vegetation: A meta‐analysis. Journal of Ecology, 87, 984–994. 10.1046/j.1365-2745.1999.00414.x

[ece36351-bib-0063] Zobel, M. (1997). The relative of species pools in determining plant species richness: An alternative explanation of species coexistence? Trends in Ecology & Evolution, 12, 266–269. 10.1016/s0169-5347(97)01096-3 21238064

[ece36351-bib-0064] Zobel, M. (2016). The species pool concept as a framework for studying patterns of plant diversity. Journal of Vegetation Science, 27, 8–18. 10.1111/jvs.12333

[ece36351-bib-0065] Zuur, A. F. , Ieno, E. N. , & Elphick, C. S. (2010). A protocol for data exploration to avoid common statistical problems. Methods in Ecology and Evolution, 1, 3–14. 10.1111/j.2041-210X.2009.00001.x

